# Role of the IL23/IL17 Pathway in Crohn’s Disease

**DOI:** 10.3389/fimmu.2021.622934

**Published:** 2021-03-30

**Authors:** Heike Schmitt, Markus F. Neurath, Raja Atreya

**Affiliations:** ^1^ First Department of Medicine, Friedrich-Alexander-University Erlangen-Nürnberg, Erlangen, Germany; ^2^ Deutsches Zentrum Immuntherapie, Erlangen, Germany

**Keywords:** Crohn’s disease, anti-TNF therapy, IL17/IL23 axis, intestinal immunity, inflammation, resistance to apoptosis, non-responder

## Abstract

Crohn’s disease (CD) is a chronic relapsing disorder of the gastrointestinal tract and represents one of the main entities of inflammatory bowel disease (IBD). CD affects genetically susceptible patients that are influenced by environmental factors and the intestinal microbiome, which results in excessive activation of the mucosal immune system and aberrant cytokine responses. Various studies have implicated the pro-inflammatory cytokines IL17 and IL23 in the pathogenesis of CD. IL23 is a member of the IL12 family of cytokines and is able to enhance and affect the expansion of pathogenic T helper type 17 (Th17) cells through various mechanisms, including maintenance of Th17 signature genes, upregulation of effector genes or suppression of repressive factors. Moreover, IL17 and IL23 signaling is able to induce a cascade of pro-inflammatory molecules like TNF, IFNγ, IL22, lymphotoxin, IL1β and lipopolysaccharide (LPS). Here, IL17A and TNF are known to mediate signaling synergistically to drive expression of inflammatory genes. Recent advances in understanding the immunopathogenetic mechanisms underlying CD have led to the development of new biological therapies that selectively intervene and inhibit inflammatory processes caused by pro-inflammatory mediators like IL17 and IL23. Recently published data demonstrate that treatment with selective IL23 inhibitors lead to markedly high response rates in the cohort of CD patients that failed previous anti-TNF therapy. Macrophages are considered as a main source of IL23 in the intestine and are supposed to play a key role in the molecular crosstalk with T cell subsets and innate lymphoid cells in the gut. The following review focuses on mechanisms, pathways and specific therapies in Crohn’s disease underlying the IL23/IL17 pathway.

## Crohn’s Disease

CD representing one of the major forms of inflammatory bowel diseases (IBD), is a chronic inflammatory condition affecting the gastrointestinal tract (1). The global annual incidence of IBD is rising and it is estimated that the incidence of IBD in European countries is 3-8.5/100,000, and as many as 2.2 million people in Europe suffer from IBD (2). All parts of the gastrointestinal tract can be affected whereas the terminal ileum and the colon are the most frequent localizations (3). CD is thought to be the result of the interaction between genetic susceptibility, environmental factors and the intestinal microflora causing abnormalities in mucosal immune response and altered epithelial barrier function (1, 4). CD is associated with significant morbidity and has a marked impact on the patient’s quality of life as the most common symptoms include abdominal pain, diarrhea, rectal bleeding, weight loss, fever, and fatigue. Extra-intestinal inflammation manifests frequently in the eyes, liver, skin and joints, reflecting the systemic nature of this debilitating disease. Moreover, the majority of patients eventually develop penetrating or stricturing complications leading to repeated surgeries and disability (5, 6). The pathogenesis of CD is complex. Recent studies have greatly improved our understanding of the pathophysiology of CD, leading to major advances in the treatment and diagnosis of CD (7, 8). Earlier treatment goals focused on reducing clinical symptoms, but in the course of time and the development of new-targeted therapies, the initial goal of achieving clinical remission, shifted to steroid-free remission, endoscopic remission and mucosal healing, which have all become an integral part of successful CD treatment (9, 10). The first class of substances approved for the treatment of CD were anti-TNF antibodies (infliximab, adalimumab and certolizumab pegol). In the next few years, antibodies against the integrin alpha4beta7 (vedolizumab) and interleukin 12 (IL12) and interleukin 23 (IL23) through their common p40 subunit (ustekinumab) have been approved for CD therapy (11, 12). Moreover, recently published data demonstrate that the treatment with the selective IL23p19 inhibitors risankizumab or brazikumab leads to high response rates in CD patients that did not respond to previous anti-TNF therapy (13, 14). Although the aforementioned-targeted therapies have achieved great clinical success, it was found that only a subgroup of CD patients benefit from these treatments. In addition, there are currently no clinically compatible predictive biomarkers for individual guidance of drug therapy. Therefore, it is of utmost clinical importance to gain a deeper understanding of the respective modes of action of each therapeutic substance class to ensure that each patient is provided with the most effective and appropriate therapy (15, 16).

## IL23 Signaling

**Figure 1:**
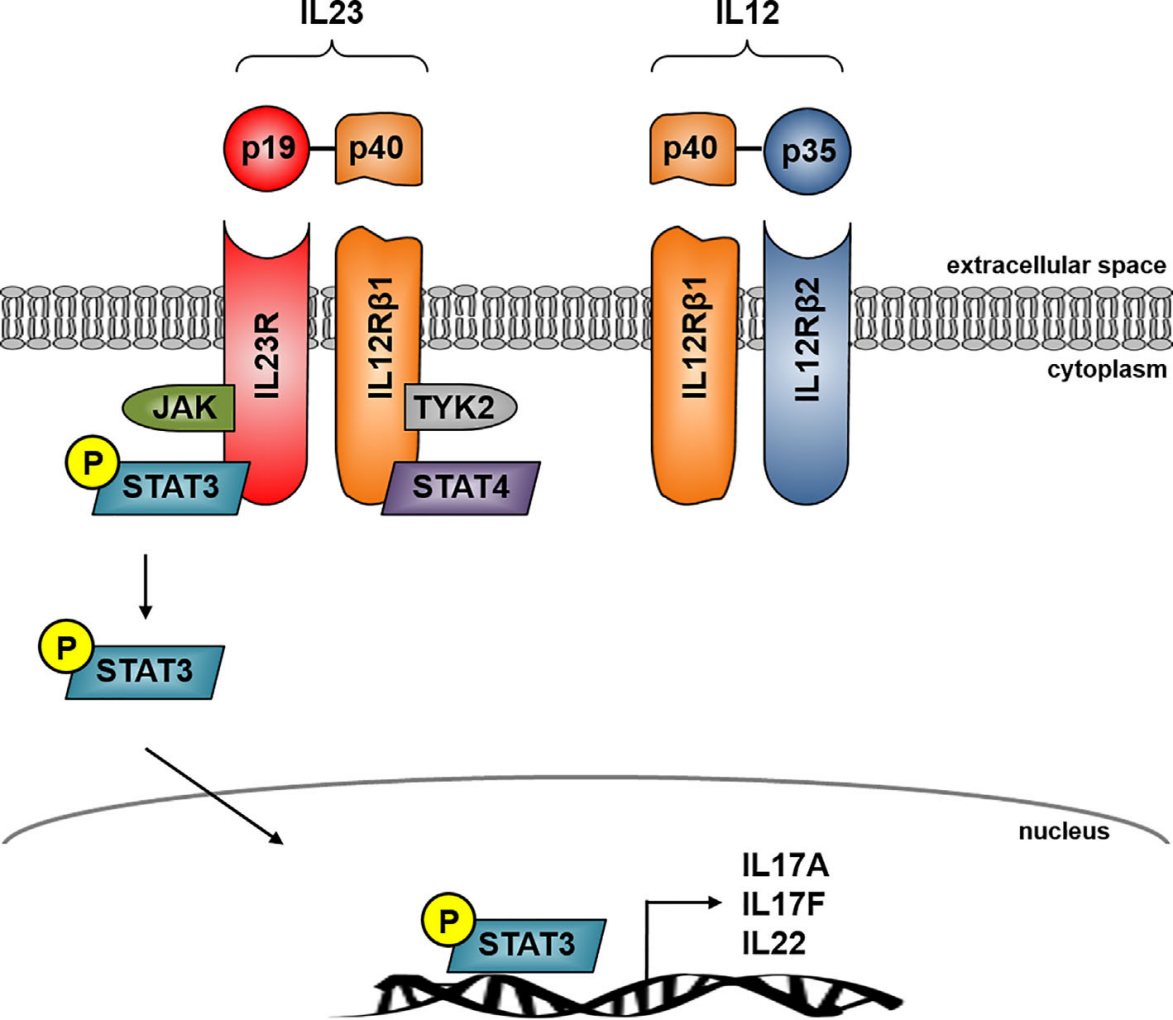
IL23 signaling in Crohn’s disease. IL23 is a heterodimer consisting of the unique subunits p19 and p40, the latter is shared with IL12. IL23 signals through its heterodimeric receptor complex consisting of the two subunits IL12Rβ1 and IL23R whereas IL23R is the unique subunit and IL-12Rβ1 shares the IL12 receptor complex. The IL23R complex signals through JAK kinase and STAT transcription factors. IL23 binding to its receptor activates Jak2 and Tyk2 kinases which then phosphorylates the receptor to form a docking site leading to the subsequent phosphorylation of STAT3 for the p19 subunit and STAT4 for the p40 subunit. IL23R signaling activates several pathways leading to transcription of several effector cytokine genes in CD including IL17A, IL17F and IL22.

IL23 is a heterodimer cytokine consisting of the p40 subunit (shared with IL12) and the unique p19 subunit (IL23A) encoded by the IL23 gene (17). IL23 belongs to the IL12 cytokine family whereas the human p19 is a four alpha-helix protein with 70% similarity to its mouse orthologue (18). The heterodimer cytokine IL12 is built by the two subunits p40 (shared with IL23) and p35. IL23 signals through its heterodimeric receptor complex consisting of the two subunits IL12Rβ1 and IL23R, while IL12 signals through its heterodimeric receptor complex consisting of the two subunits IL12Rβ1 and IL12Rβ2. The shared p40 subunit of IL12 and IL23 signals through IL12Rβ1 whereas the unique subunit IL23p19 signals through IL23R and the unique IL12p35 interacts with IL12Rβ2 (19) (**Figure 1**).

IL23 binding to its receptor activates Janus kinase 2 (jak2) and tyrosine kinase 2 (tyk2), which then phosphorylates the receptor to form a docking site leading to the subsequent phosphorylation of signal transducer and activator of transcription 3 (STAT3) for the p19 subunit and STAT4 for the p40 subunit. The initiation of IL23R signaling leads to the activation of several pathways, which are centrally involved in the pathogenesis of CD, for example P38 MAPK, PI3K-Akt or the NFкB pathway. This activation leads to the release of CD associated cytokines like IL17A, IL17F or IL22 (20–22) (**Figure 1**).

## IL23 in Crohn’s Disease

**Figure 2:**
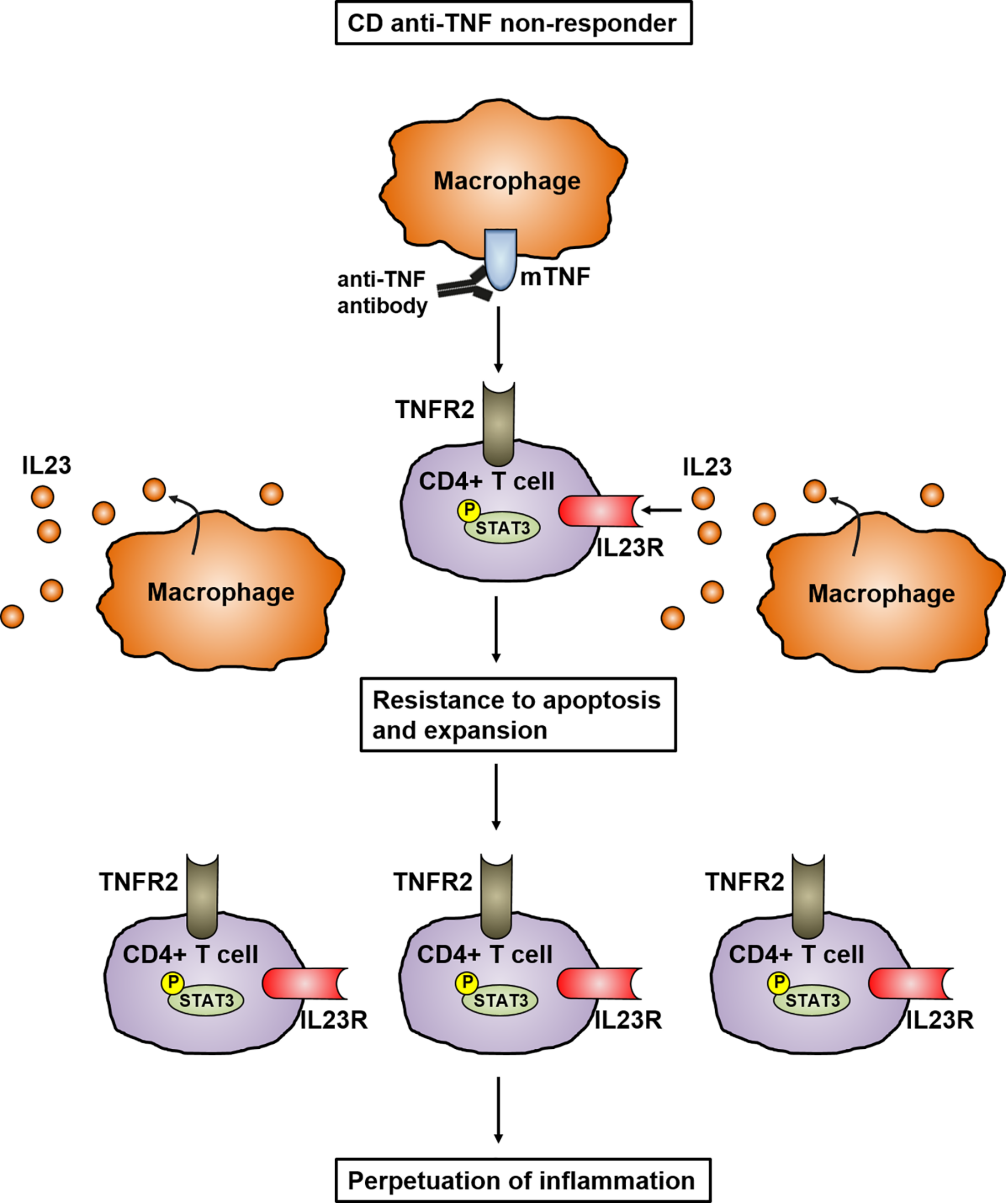
Model of IL23 mediated resistance to apoptosis of mucosal CD4+ T cells in anti-TNF refractory Crohn’s disease patients. In anti-TNF refractory patients, TNFR2 bearing gut CD4+ T cells express the IL23R. Heightened production of IL23 from CD14+ macrophages leads to binding to the IL23R on CD4+TNFR2+ T cells and induction of STAT3 activation. This activation leads to the expansion of CD4+IL23R+TNFR2+ T cells that are resistant to apoptosis induction by anti-TNF antibodies, resulting in the perpetuation of mucosal inflammation.

Different studies have shown that a multitude of cytokines play an important role in the development and perpetuation of CD. It has been proven that IL23 in particular is mainly involved in the pathogenesis of CD (23, 24). Genome-wide association study (GWAS) have analyzed the polymorphism in the gene encoding IL23R and linked it to the pathogenesis of IBD, indicating the important role of IL23 in mucosal inflammation. In addition, the elevated levels of IL23 in the mucosa of CD patients further emphasizes its key role in the pathogenesis of IBD (25). IL23 is mainly expressed by CD14+ intestinal macrophages that are key players in mediating the perpetuation of inflammation by infiltrating into the inflamed intestine in CD patients (26–28). Dendritic cells and epithelial cells were also shown to produce IL23 (29). This is supported by a recently published study showing that mucosal TNFR2-expressing CD4+ T cells circumvent anti-TNF-induced apoptosis by coexpressing IL23R, which is activated by the upregulated IL23 production of mucosal CD14+ macrophages. Here, IL23 caused the activation of pSTAT3 in CD4+ mucosal T cells, which results in resistance to apoptotic signals. The activated T cells are characterized by the release of high amounts of Th1 and Th17 cytokines. These TNFR2+IL23R+T cells expand and accumulate in the mucosa of anti-TNF-refractory CD patients, where they perpetuate chronic intestinal inflammation (28) (**Figure 2**). These data imply that anti-TNF resistant patients could benefit from therapies specifically targeting IL23.

## The Role of IL23 in the Development of Th17 Cells

**Figure 3:**
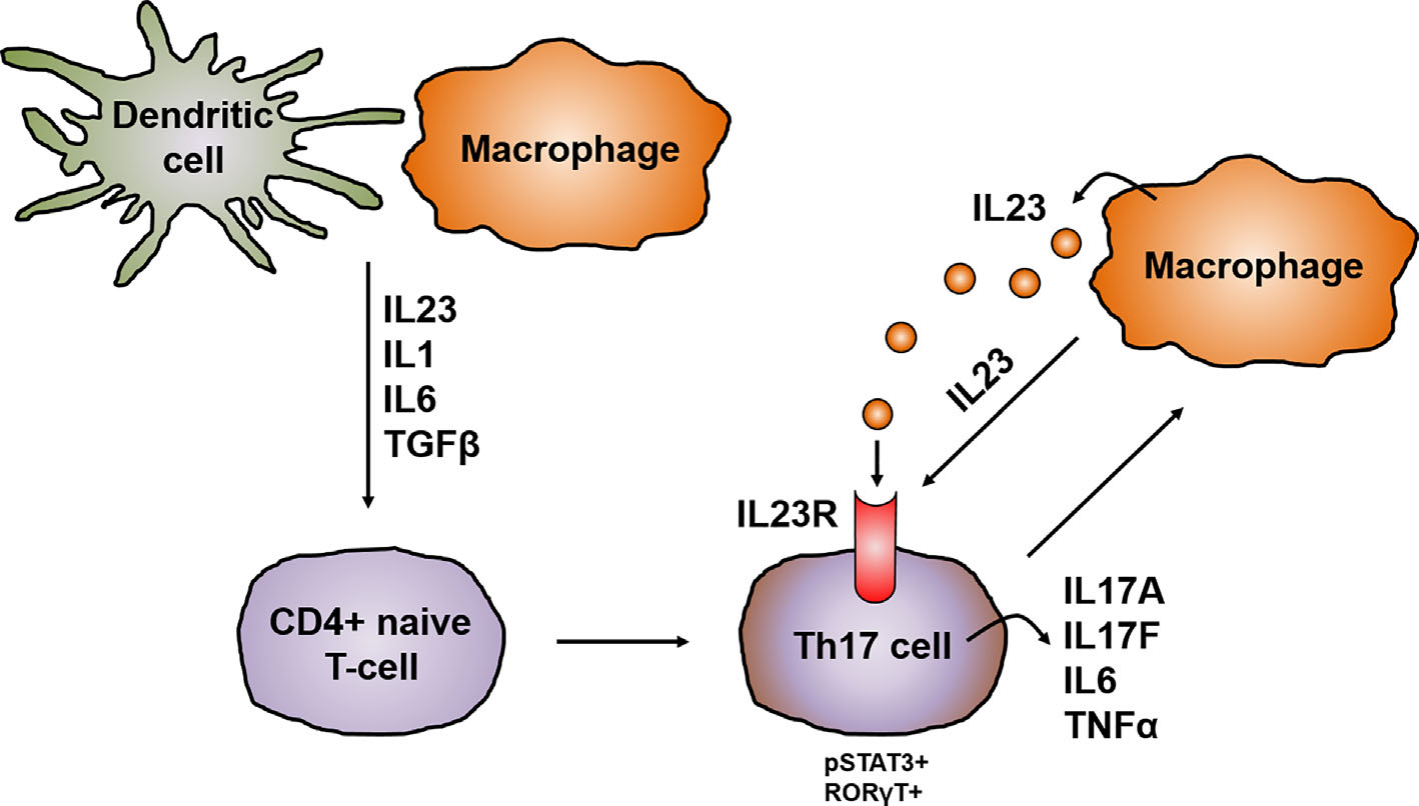
IL23 in the development and activation of Th17 cells. In chronic inflammation, antigen-presenting cells like dendritic cells and macrophages are the main producers of IL23, which promotes together with other cytokines like IL1, IL6 and TGFβ the development of IL17 producing pathogenic Th17 cells. The differentiation of Th17 cells is prompted by the synergistically working of STAT3 and RORγt leading to the upregulation of the IL23R on Th17 cells and the release of other pro-inflammatory cytokines like IL17A, IL17F, IL6 or TNFα. This in turn leads to the production of IL23 mainly by macrophages. IL23 is on the one hand important for the maintenance and expansion of the Th17 lineage and in addition acts mainly on macrophages in an autocrine manner.

CD4+ helper T cells are pivotal players in the pathogenesis of CD and, depending on the cytokine milieu, differentiate into regulatory and effector T cells i.e. Th1, Th2, Th17, follicular helper T cells (Tfh) and regulatory T-cells (Tregs). Until the discovery of other T cell lineages, Th1 and Th2 were longtime considered to be the only cells arising from progenitor CD4+ helper T cells (30). The Th1/Th2 paradigm offered a framework for understanding the pathogenesis of IBD and several other chronic inflammatory diseases. However, the distinguishing proof of Th17 cells has greatly extended the understanding of autoimmunity and inflammation and provided missing scientific links that could not be solely explained by Th1 and Th2 cells. Specific signal transduction mechanisms, several transcription factors and milieu specific cytokine patterns are responsible for the polarization of progenitor CD4+ helper cells (31). Distinct from the development of Th1 and Th2 cell lineages, Th17 cell differentiation is prompted by the synergistic work of STAT3 and the transcription factor retinoid acid related-orphan nuclear receptor gamma (RORγt). The activation of RORγt causes the expression of IL17 and IL23 receptor (IL23R), leading to the production of IL23 by various immune cells, like dendritic cells or monocytes/macrophages, which in return increases the expression of RORγt and IL17 *via* STAT3 (32). The IL23R is absent on naïve CD4+ helper T cells leading to the idea that IL23 alone is not able to induce Th17 cell development. Indeed, it was shown that IL23 is especially important for maintenance and expansion of the Th17 lineage *via* a positive feedback loop that upregulates IL17, RORγt, TNF, IL1 and IL6. This positive feedback is centrally involved in the expansion of pathogenic pro inflammatory Th17 cells in CD (33–35) (**Figure 3**).

## Th17 Cells and IL17 in the Pathogenesis of Crohn’s Disease

The IL17 cytokine family consists of six ligands, IL17A to IL17F and is the key cytokine produced by Th17 cells. Besides IL17, Th17 cells also produce IL21, IL22, IFNγ and TNF (36). The discovery of the IL23/Th17 pathway paved the way for a better and deeper understanding of the pathogenesis of CD and the involved immune cells leading to the successful development of novel therapeutic substance classes targeting this specific pathway (37). Several studies revealed that IL17 producing cells mainly accumulate in the submucosa and muscularis propria of CD patients (38). Flow cytometric analysis of mucosal cells further demonstrated the increase of IL17 producing T cells in CD patients compared to controls. Interestingly, some of these cells also coexpressed IFNγ, a more Th1 related cytokine. Subsequent stimulation of these cells with IL12 elevated the expression of the Th1 related markers Tbet and IFNγ and decreased the Th17 related markers RORγT and IL17. These results clearly indicate that IL17 producing T cells from CD patients can be polarized from Th1 cells (39, 40). Animal models have also been used to evaluate the role of Th17 cells in the pathogenesis of IBD. Zhang and colleagues could demonstrate by using IL17RA knockout mice in a trinitrobenzenesulfonic (TNBS) induced colitis model that IL17 is essential for the development of colonic inflammation. Accordingly the application of the IL17RA IgG1 fusion protein in mice with TNBS-colitis significantly decreased colonic inflammation and protected the mice from weight loss (41). Studies in the dextran-sulfate sodium (DSS)-induced colitis model revealed that IL17F deficiency leads to colitis reduction, whereas IL17A deficiency resulted in a more severe course of the disease (36, 42, 43). In line with this, a monoclonal antibody against IL17A (secukinumab) failed to show therapeutic efficacy in the treatment of CD, moreover a high rate of adverse events and increased severity of the disease compared to the placebo group was reported (43).

## Th17 plasticity and Its Relevance in Chronic Inflammation

Polarized T cells have the ability to change their phenotype and repolarize towards various fates. This innate flexibility is termed plasticity (44). The plasticity of cells can be influenced by several factors like the cytokine environment, metabolites or different microbial components. The cytokine milieu drives T cell subset development and also induces plasticity through the activation of distinct and specific STAT molecules and multiple transcription factors like FOS-like antigen (Fosl2) or interferon regulatory factor (IRF4) (45, 46). The plasticity of Th1-Th17 has been reported to play an essential role in the regulation of intestinal immune responses (47). Several studies indicate that the development of IBD is associated with both Th1 and Th17 cells. The accumulation of Th1 and Th17 cells in the mucosa of IBD patients results in elevated IFNγ and IL17 levels compared to healthy controls. IFNγ+ IL-17+ co-expressing cells are considered to be Th17 cells that transform into Th1 lymphocyte progenitor cells, demonstrating the important role of Th17/Th1 plasticity in the pathogenesis of chronic intestinal inflammation (48). IL23 signaling can drive the conversion of Th17 to Th1 cells by shifting the secretion of IL17A to IFNγ *in vivo* (49). Here, IL23 may suppress IL17 expression and enhance IFNγ release through a STAT4/T-bet-dependent pathway, particularly under conditions of decreased TGFβ expression, a sustained inducer of IL17A and IL17F (50). Moreover, a murine model with CD4+ T cells lacking the IL23R has revealed that IL23R signaling induces colitis, associated with the induction of IFNγ and IL17A co-expressing cells (51). Interestingly it was also shown that Th17 derived Th1 cells express CD161, which is a surface marker on Th17 cell progenitors (52). Studies have also demonstrated that IFNγ+ IL17+ coexpressing T cells from CD patients express the IL23R and therefore are centrally involved in the pathogenesis of CD (28). Therapies targeting IL17, IFNγ or IL23 might therefore also have an influence on Th17-dervied Th1 cells. The above described research findings demonstrate that Th17/Th1 cells play a pivotal role in the development and pathogenesis of IBD.

## IL23 and IL17 Responsive Cells

Different studies have demonstrated that the interaction with IL23 and its receptor mainly leads to phosphorylation of STAT3, building up a positive feedback loop that triggers gene expression important for Th17 cell activation and effector functions (53). IL23 is essential for the maturation and expansion of Th17 cells in humans and mice and is indispensable for their initial differentiation from naive CD4+ T cells to fully pathogenic Th17 cells (54). These Th17 cells massively infiltrate the inflamed intestine of CD patients, where they produce pro-inflammatory cytokines like IL17 and thereby perpetuating the inflammatory process (55). Besides Th17 cells, a variety of innate immune cells respond to IL23, including subsets of γδ T cells, natural killer T (NKT) cells, intrathymically primed “natural” Th17 cells and innate lymphoid cells (ILC) (54). These innate immune cell subsets are collectively referred to as “type 17 cells” and are located in non-lymphoid organs where they are able to respond immediately to tissue damage or pathogen invasion. Stimulation of Th17 cells and type 17 cells with IL1β and IL23 induces local tissue inflammation, characterized by type 17 signature cytokines such as IL17, IL22 and GM-CSF (56). Furthermore, it was shown that IL23 is able to induce IL17 expression in RAG – deficient mice (lacking B and T cells), demonstrating that innate IL17 producing cells are an integral part in IL17 based immune responses (57). Several publications indicate that these IL23 dependent innate IL17 producing cells are mainly found in the skin and mucous membranes where they play a central role in homeostasis (58–60).

ILC3 cells express the transcription factor RORγt and are important players in protecting against extracellular pathogens in the gastrointestinal mucosa. IL23 responsive ILCs are located in human mucosa-associated lymphoid tissue, for example the intestinal Peyer’s patches (59). ILC3 cells are considered to be responsible for gastrointestinal mucosal homeostasis in the physiological state through moderate production of IL22, IL17, and GMCSF. A dysregulation of ILC3 cells cause the overexpression of the inflammatory cytokines IL22 and IL17. Subsequently, neutrophils are recruited and cleave epithelial cadherin and junctional adhesion molecule-like molecules (JAMLs), resulting in elevated epithelial permeability (61). Moreover, these cells have also been linked to the pathogenesis of IBD, as they express the IL23R, leading to overproduction of several effector cytokines like IL12, IFNγ and IL17 by these cells in an IL23 depending manner (62, 63). In line with these data, Geremia and colleagues could demonstrate that IL23 responsive ILCs accumulate in the mucosa of CD patients where they produce inflammatory cytokines leading to intestinal inflammation (23).

γδ T cells are mainly found in mucosal and skin surfaces, more precisely in the intestinal intraepithelial compartment, and also show a broad expression of IL23R. They play a central role in the mucosal barrier due to their expression of pattern recognition receptors (PRRs) such as CLEC7A or TLR2 (64). Since peripheral γδ T cells are capable of recognizing both self- and non-self-ligands, it is assumed that these cells can be separated into two main categories of “antigen-experienced” and “antigen-naïve” γδ T cells (65). Recently, studies have demonstrated that γδ T cells are key innate IL17-producing cells in autoimmune inflammation and infectious diseases (66, 67). After stimulation with IL23, γδ T cells start to secrete IL22, IL21 and IL17. Their role in the pathogenesis of CD is not fully understood but studies in several mouse colitis models suggest an important role of γδ T cells in this context and also in other chronic inflammatory diseases (68).

CD1d- expressing NKT cells are mainly found in the human intestine where they recognize lipids from commensal microbes. Based on their T cell receptor (TCR) characteristics, NKT cells are stratified into two main subsets, type I and type II NKT cells (69). They are centrally involved in the regulation of intestinal homeostasis and inflammation (70). After stimulation with IL23, NKT cells produce large amounts of IL22 and IL17. Several murine colitis models have indicated that the contribution of NKT cells can be protective or pathogenic. Here, the kind of inflammatory stimuli and lipid antigens play a crucial role in determining the immune response (69). Various clinical studies indicated reduced levels of type I NKT cells in the intestine and peripheral blood of CD and UC patients (71, 72). In contrast, another study revealed an accumulation of type II NKT cells in the lamina propria of UC patients (73).

Thus, the discovery of the IL23/IL17 pathway has led to fundamental changes in our understanding of cellular immunity and essentially contributed to the development of clinical trials and therapeutic strategies targeting the IL23/IL17 pathway in CD.

## The Impact of IL23R Polymorphism on Th17 Cell Function

GWAS studies have revealed more than 200 risk variants associated with IBD, most of them affect CD and UC. The majority of disease-related single nucleotide polymorphisms (SNPs) occur in non-coding regions of the genome (74, 75). Interestingly, the variants in the IL23R are protein-coding and are therefore an exception in contrast to the large portion of non-coding risk variants. In 2006, a study by Duerr and colleagues revealed a link between variants of the IL23R gene on chromosome 1p31 and ileal Crohn’s disease (24). Especially the coding variant R381Q has been linked with functional consequences to T cell immunity. CD patients carrying the protective variant of the IL23R produce reduced levels of IL17 and IL22 after IL23 stimulation, resulting in lower frequencies of circulating Th17 cells (76). It could further be shown that T cells from these patients display a diminished IL23 mediated phosphorylation of STAT3 and release less IL17 after exposure to *Borrelia burgdorferi*, a strong inducer of Th17 responses (77). A case-control study with 201 CD patients demonstrated that the development of CD is associated with the IL23R variant G149R (78). In contrast, further studies noted by using a candidate gene approach that SNPs in IL23R leads to high activation of the IL23/IL17 pathway, which was also linked with increased risk for CD and UC (77). These insights in the gene polymorphism of IL23R also affects the strategy of treatment. It was shown that IL23R genotype status determine early response to infliximab (79). Taken together, the recent years of research suggest that disease protective variants of the IL23R are more associated with reduced IL23R activity, whereas disease associated variants are more linked to elevated IL23R signaling.

## Therapeutic Approaches Targeting IL23 and IL17 Signaling

The recent finding of the critical role of IL23 and IL17 in the pathogenesis of IBD and other immune-mediated diseases has led to the development of new therapeutic approaches targeting these cytokines and corresponding receptors (56, 80, 81). First studies were conducted with anti-p40 antibodies (the shared subunit of IL23 and IL12) such as briakinumab (82) or ustekinumab (83). In another study, ustekinumab treated CD patients with a moderate to severe disease course displayed an increased rate of response and remission to ustekinumab induction and maintenance treatment compared to the placebo treated group (12, 84). Anti-TNF treated CD patients with severe psoriasisform lesions and dermal Th17 cell infiltrates were additionally treated with ustekinumab, leading to a remarkable suppression of skin lesions (85). The promising results of ustekinumab treatment emphasizes the important role of the interaction of IL23/IL23R and IL17/IL17R in the pathogenesis of CD. The blockade of the selective IL23p19 subunit (which is not shared with IL12) allows normal Th1 responses that are mediated by IL12. In contrast to directly antagonizing IL17 function, an IL23 blocking antibody should inhibit the IL23 dependent development and proliferation of pathogenic Th17 cells, which subsequently leads to the reduction of pro-inflammatory cytokines associated with this cell type, such as IL17, IL21 and IL22. Based on the clinical efficacy of IL23 specific inhibitors in psoriasis, more recent studies evaluated the effects of IL23p19 blockade in CD. Risankizumab is a humanized monoclonal antibody targeting the p19 subunit. In a phase 2 trial, 121 patients with active CD were randomized to receive different doses of risankizumab or placebo. After 12 weeks, a significantly higher proportion of patients, which were treated with 600mg risankizumab, achieved clinical remission in comparison to the placebo group. Analysis of mucosal samples revealed that risankizumab treatment leads to the suppression of various genes linked to the IL23/IL17 axis (13, 86, 87). The treatment of risankizumab also leads to the maintenance of remission at week 26 in treated CD patients (86). Brazikumab, another p19 blocker, is a fully human IgG2 IL23 antibody and was tested in a phase 2 study with active CD patients that failed previous anti-TNF therapy (14). In this study, clinical improvement of CD patients 8 and 24 weeks after initiation of brakizumab therapy could be achieved in comparison to the placebo treated group. In addition, patients receiving brazikumab had greater reductions in serum IL22 levels than placebo treated patients, again emphasizing the importance of the IL23/IL17 axis in the pathogenesis of CD (14). Here, patients with elevated baseline IL22 serum levels had a higher probability of achieving clinical remission upon brazikumab treatment.

Further late-stage clinical studies targeting p19 are currently being conducted (e.g. with the p19 neutralizing antibodies risankizumab (13), brazikumab (14), mirikizumab (88) or guselkumab (89)). The p19 antibody tildrakizumab has not yet been tested in CD patients, but has proven therapeutic efficacy in phase 3 trial in psoriasis patients (90). The oral peptide PTG-200 that selectively antagonizes the IL23R was well tolerated in a phase 1 trial in healthy volunteers (91) and will be tested in CD phase 2 trials. A summary of the pharmaceutical compounds can be found in **Table 1**.

**Table 1:** Targeted therapies directed against IL12, IL17, IL23 or their respective receptors.

Drug	Route	Target	Current stage of development
Ustekinumab	IV/SC	p40	Approved for induction and maintenance therapy (12)
Risankizumab	IV/SC	p19	Phase 2 study (13)
Brazikumab	IV/SC	p19	Phase 2a study (14)
Mirikizumab	IV/SC	p19	Phase 2 study (88)
Guselkumab	SC	p19	Phase 2 study (89)
Briakinumab	IV/SC	p19	Phase 2b study (82); did not meet primary endpoint, halted development
Tildrakizumab	SC	p19	No Crohn’s disease data
PTG-200	Oral	IL23R	Phase 1 study (91)
Secukinumab	IV	IL17	Phase 2a study (43); worsening of disease, halted development
Brodalumab	IV	IL17R	Phase 2a study (92); worsening of disease, halted development

IV = intravenous; SC = subcutaneous.

**Figure 4:**
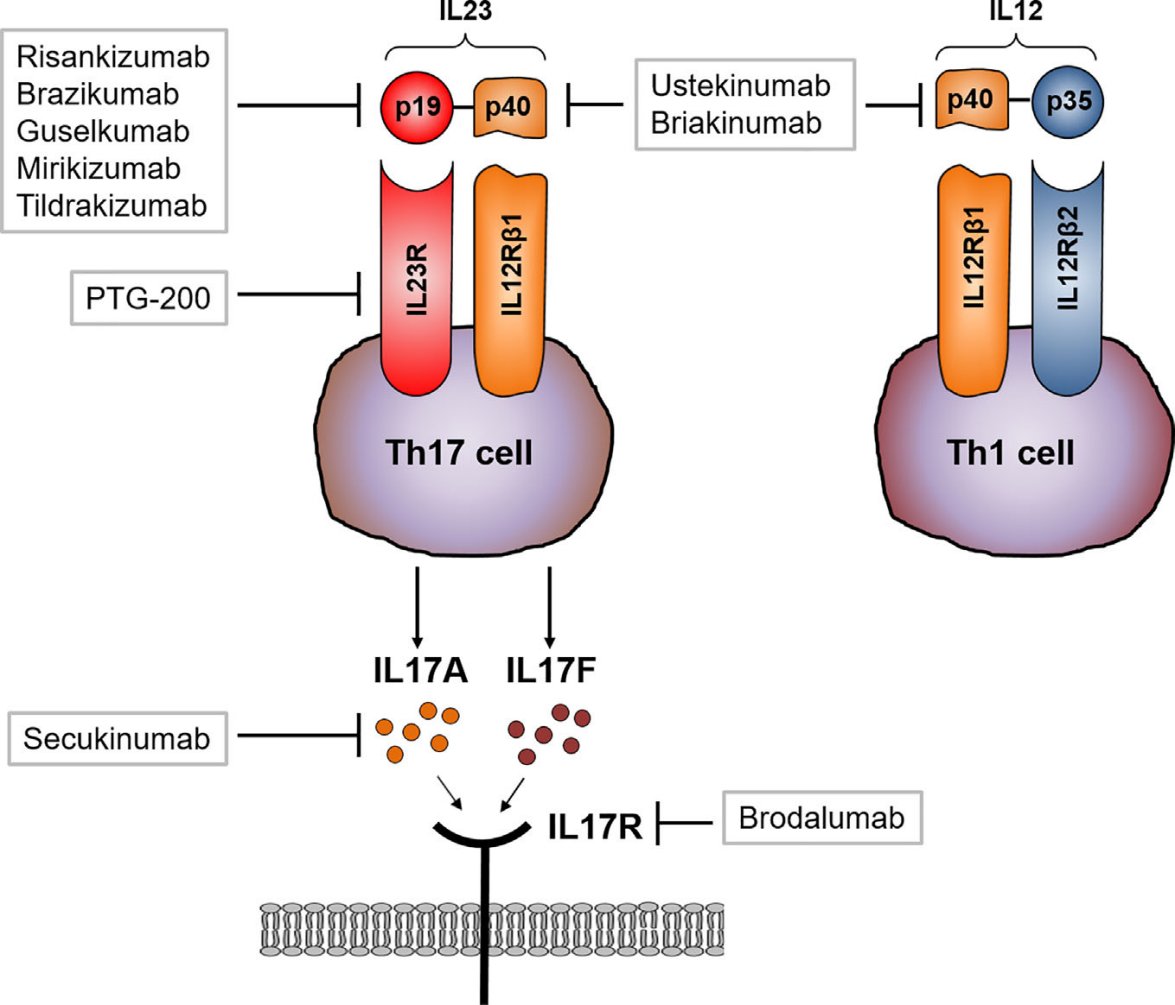
Therapeutic approaches targeting IL23 and IL17 signaling. Ustekinumab and briakinumab specifically blocks the IL12/IL23 subunit p40 in CD patients whereas risankizumab, brazikumab, guselkumab and mirikizumab selectively block the unique subunit p19. Activated Th17 cells produce large amounts of IL17. Secukinumab directly binds to IL17A and thereby inhibits the interaction with the IL17 receptor (IL17R). Brodalumab directly binds to the IL17R causing an inhibition of IL17 ligand binding (A and F) to their receptor.

As mentioned above, blocking IL17 signaling directly in CD patients might also influence the Th1 immune response, including microbial defense. Two different strategies blocking IL17 in CD patients with moderate to severe CD have been evaluated. Secukinumab directly targets IL17A whereas brodalumab blocks the IL17R subunit IL17RA. Secukinumab therapy did not meet the primary endpoint but rather led to worsening of disease and furthermore a heightened incidence of severe adverse such as fungal infections were reported compared to the placebo treated group (43). Similarly, brodalumab treatment in CD was prematurely stopped as numerical worsening of CD in the antibody treated group was found (92) Interestingly, both antibodies show high efficacy in the treatment of psoriasis (93–95) (**Figure 4**).

In contrast to IL23, different murine models of colitis suggest a protective role for IL17A. It was shown that the neutralization of IL17A in a dextran sodium sulfate (DSS) murine colitis model resulted in elevated tissue damage (96) and T cells, lacking IL17A or IL17R, transferred into RAG-1 deficient mice, led to increased severity of the colitis course (97). Interestingly, it was further demonstrated that IL17A is able to promote epithelial barrier function by regulating proteins like occluding, which is an important tight junction protein. This protection leads to less excessive gut permeability after epithelial injury in a colitis mouse model (98). In this study, colonic IL23R+ γδ T cells were the main producers of gut-protective IL17A. Moreover, the protective effect of IL17 was also present in the absence of IL23, indicating an IL23 independent release of protective IL17A from IL23R+ γδ T in this context (98). While several studies clearly could not demonstrate any efficacy for neutralizing IL17A or IL17RA in CD, the current understanding of the mechanism of IL17 mediated protective effects in both mouse and man is still elusive.

## Janus Kinase (JAK) Inhibitors in IBD

Most pathways that are involved in IBD are characterized by the massive production of pro-inflammatory cytokines by different immune cells, leading to the inflammation of the mucosa or the disruption of the intestinal barrier (99). JAKs are cytoplasmic tyrosine kinases that transform extracellular processes into various intracellular immune and inflammatory processes (100). One central role of cytokines is the contribution to transcellular signaling by activating the JAK signal transducer and activator of transcription JAK/STAT pathway (101). The IL23 signaling pathway includes the activation of members of the JAK family of tyrosine kinases and the several downstream transcription factors of the STAT family. IL23R signaling is linked to Jak2 and Tyk2 leading to the phosphorylation of STAT3 (**Figure 1**).

JAK inhibitors influence several inflammatory pathways and are therefore a promising target for inflammatory diseases like IBD. However, blocking JAKs in CD or UC patients showed contradictory results (102).

Tofacinitib is a pan-JAK inhibitor that demonstrated efficacy in patients with moderate to severe UC (103). In contrast, Tofacitinib has not reached the primary endpoint in CD patients leading to the discontinuation of clinical trials for the treatment of CD patients with Tofacitinib (104, 105).

Filgotinib has a 28-fold more selectivity for JAK1 compared with JAK2 and is therefore regarded as a JAK1 inhibitor (106). The efficacy of Filgotinib for the induction of remission in moderate to severe CD patients was evaluated in the randomized, placebo-controlled, multicenter phase II study (107) and showed promising efficacy data.

Upadacitinib is an oral JAK1 selective inhibitor with a 74-fold more selectivity for JAK1 over JAK2. The efficacy of Upadacitinib for the induction and maintenance of remission in moderate to severe CD patients was studied in a randomized, placebo-controlled multicenter phase II trial (108) and similarly demonstrated convincing signs of effectiveness. Subsequent studies will have to clarify whether more specific JAK inhibition is able to achieve high efficacy, while providing a convincing safety profile.

Altogether, JAK inhibitors represent an attractive therapeutic category of molecules for targeting IL23 downstream. Therefore, JAK inhibitors may represent an effective treatment for IBD, although potential benefits in efficacy and safety for CD need further evaluation.

## Conclusion

The discovery of the IL23/IL17 axis has changed our fundamental understanding of the pathology of chronic inflammatory diseases like CD and described a new way of how immune responses can trigger intestinal tissue damage. Until the discovery of other T cell lineages, Th1 and Th2 were longtime considered to be the only cells arising from progenitor CD4+ helper T cells. It was shown that IL23 is especially important for maintenance and expansion of the Th17 lineage *via* a positive feedback loop that upregulates IL17, RORγt, TNF, IL1 and IL6. This positive feedback is centrally involved in the expansion of pathogenic pro inflammatory Th17 cells in CD. GWAS have analyzed the polymorphisms in the gene encoding IL23R and linked it to the pathogenesis of IBD, indicating the important role of the IL23/IL17 axis in mucosal inflammation.

The current availability of the specific anti-p40 antibody ustekinumab and the expected arrival of specific anti-p19 antibodies broaden our therapeutic armamentarium in the treatment of Crohn’s disease, but inevitably leads us to the questions which patients would likely benefit the most from these compounds. Clinical trial results have indicated that prior exposure to anti-TNF therapy seems to be associated with lesser probability of responding to subsequent ustekinumab therapy in comparison to anti-TNF naïve patients (12). We still await data regarding respective effectiveness of p19 inhibitors in primarily anti-TNF naïve patients, but upregulation of the IL-23R on mucosal T cells of anti-TNF non-responders, rendering these cells more responsive to increased IL23p19 production from CD14+ mucosal macrophages, indicate the potential for anti-IL23p19-specific therapies in anti-TNF non-responders (28, 109). Recent studies have shown that the mucosal cytokine profiles shift during the course of disease (110). It could be shown that early mucosal inflammation before endoscopic recurrence showed an abundance of Th1-related cytokines and TNF and slightly increased IL17A expression in the terminal ileum. Transition from this stage to endoscopic recurrence was marked by high levels of Th1 cytokines, marked increase in IL17A, and induction of IL6 and IL23, while established lesions were characterized by a mixed Th1–Th17 profile with low levels of TNF (111). Furthermore, IL12p40 and Th1 cytokines demonstrated higher mucosal expression in recently diagnosed pediatric in comparison to patients with long-standing Crohn’s disease (112). These data might indicate that anti-p40 blockade might be particularly effective in early disease, while p19 inhibition might rather be positioned in the treatment of more established lesions. Currently conducted head-to-head trials of ustekinumab and p19 inhibitors might help us to determine the optimal place of these substances in our treatment algorithm (113). Clinical practice will also answer the important question whether patients will still benefit from anti-IL23p19 antagonism if they have previously failed to benefit from anti-IL12p40 antibody therapy, and vice versa (114). Even with the upcoming availability of p19 inhibitors in addition to the already available anti-p40 antibody, there is still the currently unmet clinical need to establish predictive markers of response to identify the subgroup of IBD patients that have a heightened probability of response to respective treatments (115). In IL23p19 inhibitors, there has so far been only a report that indicated that higher serum IL22 concentrations were associated with a greater likelihood of response to brazikumab (14). These findings must however be validated in subsequent studies and other p19 inhibitors as well before they are able to enter daily clinical practice. Only improved understanding of the mucosal immune milieu and the development of biomarkers will enable us to develop personalized approaches to treatment and future algorithms for biological therapy in these patients.

## Author Contributions

HS wrote the manuscript. MN and RA assessed the articles and their relevance to the above topics. RA supervised and drafted the manuscript and is the corresponding author. All authors contributed to the article and approved the submitted version.

## Funding

CRC1181 Project C02 (RA) and DFG-SFB/TRR241 Project No. C02 (RA) are funded by the German Research Council DFG. The German Research Council DFG funds the Heisenberg Professorship of RA. HS is supported by the Interdisciplinary Center for Clinical Research Erlangen, project J86.

## Conflict of Interest

RA has served as a speaker, or consultant, or received research grants from AbbVie, Amgen, Arena Pharmaceuticals, Biogen, Boehringer Ingelheim, Celltrion Healthcare, Dr. Falk Pharma, Ferring, Galapagos, Gilead, InDex Pharmaceuticals, Janssen-Cilag, Kliniksa Pharmaceuticals, MSD Sharp & Dohme, Novartis, Pfizer, Roche Pharma, Samsung Bioepsis, Takeda, and Tillotts Pharma. MN reports research grants and/or personal fees from Abbvie, MSD, Takeda, Boehringer, Roche, Pfizer, Janssen, Pentax and PPD.

The remaining author declares that the research was conducted in the absence of any commercial or financial relationships that could be construed as a potential conflict of interest.
